# Integrated risk factors for premature acute coronary syndrome: residual cholesterol, RDW, and BMI

**DOI:** 10.3389/fcvm.2025.1574620

**Published:** 2025-06-26

**Authors:** Yansheng Liang, Tingting Li, Jinlong Li, Haitao Han, Yanli Cheng

**Affiliations:** ^1^ Department of Cardiovascular Medicine, Binzhou Medical University Hospital, Binzhou, China; ^2^ Department of Cardiovascular Medicine, The First School of Clinical Medical of Binzhou Medical University, Binzhou, China

**Keywords:** acute coronary syndrome, residual cholesterol, red blood cell distribution width, body mass index, diagnostic value, risk assessment

## Abstract

**Introduction:**

The roles of residual cholesterol (RC), red cell distribution width (RDW), and body mass index (BMI) in premature acute coronary syndrome (ACS) remain underexplored.

**Aim:**

This study aimed to investigate the significance of RC, RDW, and BMI in the diagnosis of premature ACS.

**Methods:**

A retrospective analysis was conducted on 418 ACS patients at Binzhou Medical University Hospital, categorized into early-onset and late-onset groups. Spearman correlation and multivariate logistic regression were used to evaluate associations. Receiver operating characteristic (ROC) curves assessed the diagnostic performance of RC, RDW, BMI, and their combination.

**Results:**

RC and BMI were positively correlated with premature ACS, while RDW was negatively correlated. All three were identified as independent risk factors. A nomogram model highlighted RC as the strongest predictor. The combined model significantly improved diagnostic accuracy, achieving an area under the curve (AUC) of 0.941.

**Conclusion:**

RC, RDW, and BMI are independently associated with premature ACS, potentially reflecting inflammatory and metabolic mechanisms. Their combined use enhances diagnostic precision and may support early risk stratification in clinical practice.

## Introduction

Acute coronary syndrome (ACS) encompasses a group of clinical conditions caused by acute myocardial ischemia, including ST-segment elevation myocardial infarction (STEMI), non-ST-segment elevation myocardial infarction (NSTEMI), and unstable angina (UA). The underlying pathology typically involves atherosclerotic plaque rupture, erosion, or dissolution, followed by acute coronary thrombosis formation (1, 2). ACS is frequently the initial manifestation of cardiovascular disease and remains a leading cause of mortality among cardiovascular patients, posing a serious threat to public health (3). While the pathogenesis of ACS is multifactorial, key contributors include inflammation, dyslipidemia, and oxidative stress (4). Currently, early recognition and risk prediction of ACS rely on traditional clinical tools such as electrocardiography, serum biomarkers, and coronary imaging. However, these methods often lack sensitivity and specificity in diagnosing premature ACS, presenting a critical clinical challenge.

In recent studies, residual cholesterol (RC), red blood cell distribution width (RDW), and body mass index (BMI) have emerged as important cardiovascular biomarkers, each playing a distinct role in the pathophysiology of ACS (5–7). RC, representing cholesterol content in very-low-density and intermediate-density lipoproteins (VLDL and IDL), remains elevated despite controlled low-density lipoprotein cholesterol (LDL-C) levels. It is relevant in both fasting and non-fasting states and plays a critical role in atherosclerotic cardiovascular disease progression (8, 9). Elevated RC is known to induce chronic low-grade inflammation and is considered an independent risk factor, separate from traditional lipoproteins such as LDL-C and ApoB (10). RDW, which reflects variability in red blood cell volume, has been linked to inflammation, apoptosis, and tissue fibrosis (11). In patients with type 2 diabetes and ACS, elevated RDW not only indicates disease severity (12) but also correlates with increased cardiovascular and all-cause mortality (13). Meanwhile, BMI remains a key marker of obesity—a growing concern globally. Importantly, a recent study demonstrated that both triglyceride and BMI were independent predictors of small dense LDL-cholesterol—a key atherogenic lipid fraction—in ACS patients, and their combination significantly elevated sdLDL-C levels (14). These findings support the integration of lipid and metabolic markers for enhanced cardiovascular risk stratification. Furthermore, recent research has shown that integrated models combining inflammatory and lipid biomarkers such as RDW, BMI, and LDL-C/HDL-C outperform individual markers in predicting coronary lesion severity in ACS patients (15), further justifying a multi-indicator approach.

Despite substantial progress in understanding the individual roles of these biomarkers in cardiovascular disease, their specific contributions to premature ACS are not well established. Furthermore, existing studies largely focus on individual biomarkers rather than integrating multiple indicators into a composite model. This presents a critical gap in early diagnostic strategies. Although RC, RDW, and BMI each contribute to ACS pathogenesis and may interact biologically, their combined diagnostic potential in premature ACS has yet to be fully explored. Traditional diagnostic models, which rely heavily on symptomatic and imaging findings, often overlook the predictive value of such emerging biomarkers. This study aims to address that gap by investigating the independent and combined diagnostic value of RC, RDW, and BMI in premature ACS. These three biomarkers are not only clinically accessible and cost-effective but also reflect key physiological domains: lipid metabolism, systemic inflammation, and adiposity. By integrating them into a multifactorial predictive model, this study seeks to enhance early risk identification of premature ACS. Unlike previous research that focuses on each marker in isolation, this study evaluates their synergistic potential to improve diagnostic accuracy.

We conducted a retrospective analysis of ACS patient data from the cardiology department of Binzhou Medical University Hospital. Using advanced statistical approaches including Spearman correlation analysis, logistic regression, and receiver operating characteristic (ROC) curve analysis, we aim to develop a multifactorial model for early prediction of premature ACS. Scientifically, this study contributes to a growing body of evidence supporting the combined use of emerging biomarkers in cardiovascular risk stratification. Clinically, it offers a practical and theoretically grounded approach for early diagnosis and intervention in premature ACS, with implications for improved outcomes. Moreover, the findings may serve as a reference for integrating big data analytics and precision medicine in cardiovascular early-warning systems.

## Materials and methods

### Study design

This single-center retrospective cohort study aimed to evaluate the independent and combined diagnostic value of RC, RDW, and BMI in predicting premature ACS. Data were collected from electronic medical records of patients admitted to the Cardiology Department of Binzhou Medical University Hospital between September 2021 and March 2023. The study was based on the hypothesis that RC reflects lipid-related inflammation, RDW indicates systemic inflammatory status, and BMI reflects metabolic dysregulation—factors that may collectively contribute to premature ACS. Group comparisons, multivariate analyses, and receiver operating characteristic (ROC) curve evaluations were used to validate this hypothesis. To ensure data quality and reliability, strict quality control procedures were implemented at each stage. All laboratory parameters, including RC and RDW, were measured using standardized protocols by certified clinical laboratory technicians. Equipment calibration and internal quality control checks were performed daily in accordance with hospital regulations. BMI was calculated from height and weight measured by trained nursing staff using standardized equipment. All data extracted from the hospital electronic medical record system underwent double-entry verification by two independent researchers to minimize manual error. Inconsistencies were resolved through cross-checking with original records. Prior to statistical analysis, data were screened for outliers, missing values, and normality assumptions. Cases with incomplete or ambiguous data were excluded based on pre-defined criteria.

This study complied with the Declaration of Helsinki. Written informed consent was obtained from all participants or from a legal guardian in cases where patients were unable to consent. All data were anonymized and analyzed solely for research purposes. Data were stored on an encrypted server and destroyed upon study completion. The study protocol was approved by the Ethics Committee of Binzhou Medical University Hospital (Approval No. KT-01).

### Study population

Eligible participants were hospitalized patients diagnosed with ACS according to the 2023 European Society of Cardiology (ESC) guidelines. Diagnosis was confirmed by coronary angiography, including STEMI, NSTEMI, and UA. Patients were categorized into early-onset (<60 years) and late-onset (≥60 years) groups, based on the established age threshold for premature coronary artery disease (16). Exclusion criteria included recent use of lipid-lowering medications, severe hepatic or renal dysfunction, anemia, active infections, and other conditions that could affect the study parameters. Pregnant or breastfeeding women and patients with incomplete data were also excluded.

### Sample size description

**Figure 1:**
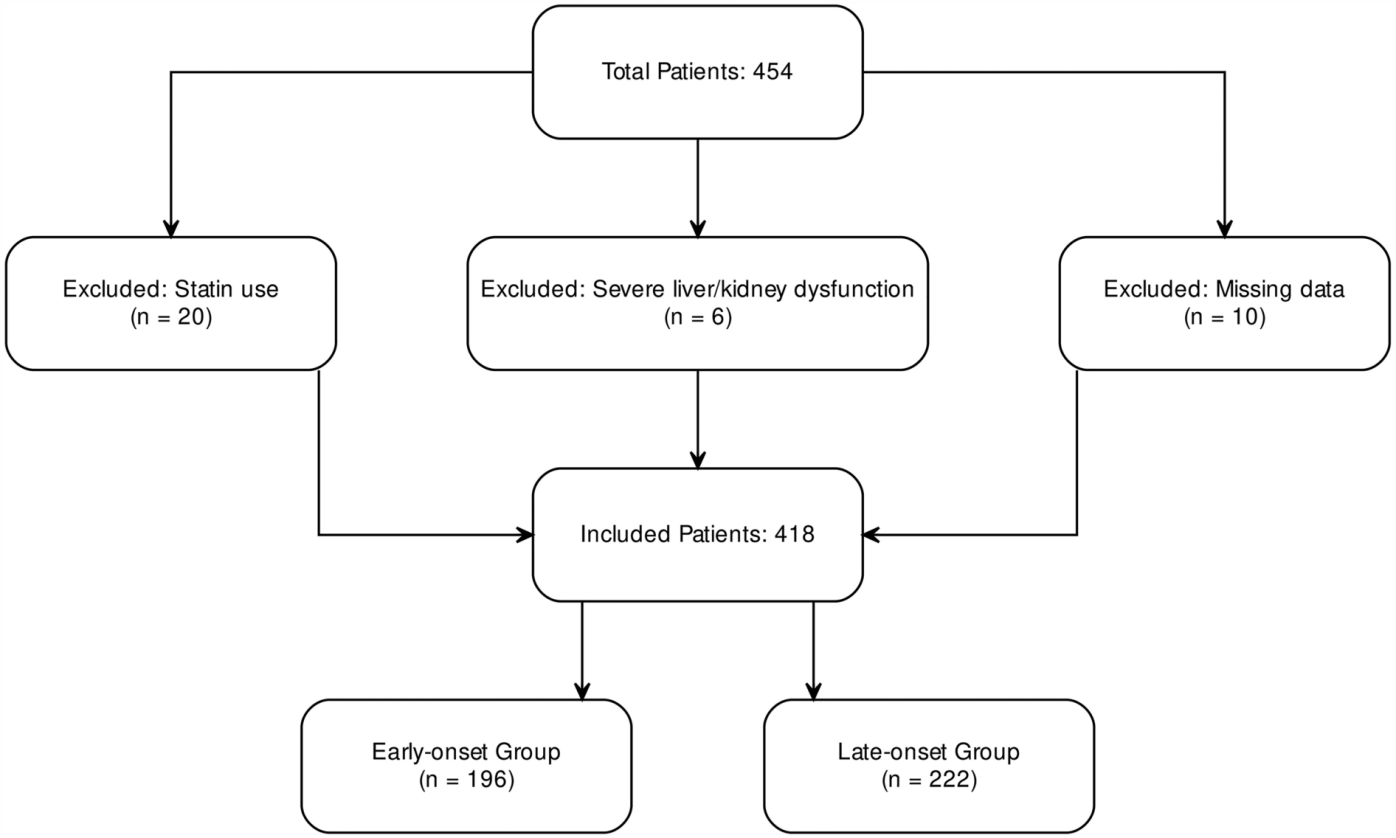
Inclusion and exclusion flowchart for ACS patients.

A total of 454 patients diagnosed with ACS were admitted to the Cardiology Department of Binzhou Medical University Hospital between September 2021 and March 2023. After excluding 36 patients due to missing critical clinical data or failure to meet inclusion criteria, 418 patients were included in the final analysis. These were categorized into an early-onset group (<60 years; *n* = 196) and a late-onset group (≥60 years; *n* = 222) (Figure 1). The sample size was determined based on the number of eligible patients during the study period and prior statistical calculations, ensuring sufficient statistical power (power >80%).

### Clinical data collection

Comprehensive data were collected for each patient, including demographics, laboratory values, BMI, and imaging findings. Demographic variables included age, sex, smoking and alcohol use, and history of hypertension and diabetes. Laboratory tests comprised white blood cell count (WBC), RDW, total cholesterol (TC), triglycerides (TG), low-density lipoprotein cholesterol (LDL-C), high-density lipoprotein cholesterol (HDL-C), and RC, calculated using the formula: RC = TC−LDL-C−HDL-C (17). BMI was calculated as weight (kg) divided by height squared (m²) (18). Imaging assessments included echocardiographic evaluation of left ventricular ejection fraction (LVEF) and coronary angiography to quantify stenosis severity using the Gensini score (19)), number of diseased vessels, and presence of calcification. All data were anonymized and securely stored for analysis.

### Comparison of study indicators

Differences in RC, RDW, and BMI were analyzed between early-onset and late-onset ACS patients. A subgroup analysis was also conducted to evaluate these markers within different ACS types, including acute myocardial infarction (AMI) and UA. This analysis revealed significant variations in metabolic and hematologic parameters between age groups, providing insights into potential pathophysiological mechanisms.

### Multivariate analysis and nomogram model construction

Pearson correlation analysis was used to examine the linear associations between RC, RDW, BMI, and premature ACS. Premature ACS was then used as the dependent variable in a multivariate logistic regression model to evaluate the independent predictive value of each marker. Based on the regression results, a nomogram was constructed to visualize the relative contribution of each variable to the risk of premature ACS, offering a tool for individualized risk prediction.

### Model performance evaluation

The predictive performance of the nomogram was assessed using the concordance index (C-index), calibration curves, and ROC curve analysis. The C-index indicated the model's discriminative ability, with values approaching 1.0 reflecting better performance. Calibration was evaluated via bootstrap resampling (1,000 iterations) to compare predicted probabilities with observed outcomes. ROC curves were generated for individual and combined predictors, and the area under the curve (AUC), sensitivity, and specificity were calculated. The combined model showed superior diagnostic accuracy compared to individual parameters.

### Statistical analysis

All statistical analyses were conducted using SPSS version 26.0 and R version 4.2.0. Normally distributed continuous variables were expressed as mean ± standard deviation (SD) and compared using independent-sample t-tests. Non-normally distributed variables were reported as median (interquartile range) and analyzed using the Mann–Whitney U test. Categorical variables were presented as counts (percentages) and compared using the chi-square test. Pearson correlation coefficients were calculated to assess linear associations. A *p*-value < 0.05 was considered statistically significant. These analyses ensured methodological rigor and robustness of the findings.

## Results

### Comparison of characteristics between early-onset and late-onset ACS patients

**Figure 2:**
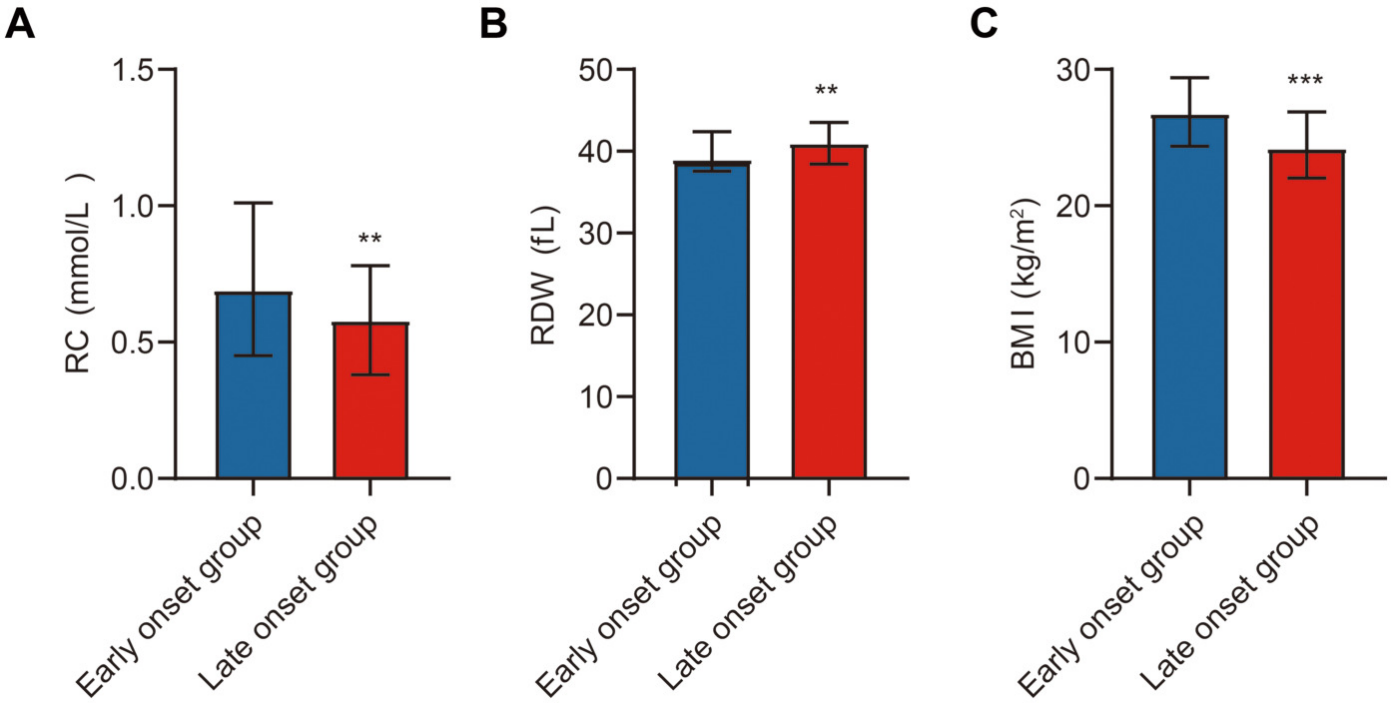
Comparison of study indicators between ACS patients. Note: **(A)** Comparison of RC between the early-onset and late-onset groups; **(B)** Comparison of RDW between the early-onset and late-onset groups; **(C)** Comparison of BMI between the early-onset and late-onset groups. Early-onset group (*n* = 196), late-onset group (*n* = 222). Statistical significance is indicated as **p < 0.01, ***p < 0.001.

We systematically compared baseline characteristics, laboratory findings, echocardiographic parameters, and study-specific indicators between early-onset (*n* = 196) and late-onset (*n* = 222) ACS patients (Table 1). No statistically significant differences were observed between the two groups in systolic blood pressure (SBP), diastolic blood pressure (DBP), heart rate (HR), smoking and alcohol history, or history of hypertension and diabetes (*p* > 0.05). However, the proportion of male patients was significantly higher in the early-onset group (*p* < 0.05). In terms of laboratory parameters, the early-onset group had significantly elevated levels of WBC, red blood cell count (RBC), hemoglobin (HGB), platelet count (PLT), alanine aminotransferase (ALT), TC, TG, and LDL-C (*p* < 0.05). Conversely, levels of NT-proBNP and D-dimer were significantly lower in this group (*p* < 0.05). No significant differences were noted in aspartate aminotransferase (AST), creatinine (Cr), blood urea nitrogen (BUN), homocysteine (Hcy), uric acid, blood glucose, HDL-C, lipoprotein(a), creatine kinase (CK), CK-MB, or troponin I (TnI) (*p* > 0.05). Cardiac ultrasound findings revealed no significant differences in left atrial (LA) and left ventricular (LV) dimensions or LVEF (*p* > 0.05). Regarding study-specific indicators, RC and BMI were significantly higher in the early-onset group, while RDW was significantly lower (*p* < 0.05) (Figure 2).

**Table 1:** Baseline characteristics of patients.

Variables	Early onset group	Late onset group	*t*/*χ*^2^/*Z*	*P* value
	(*n* = 196)	(*n* = 222)		
General information				
Male (%)	156 (79.6)	124 (55.9)	13.26	0.000
SBP (mmHg)	130.33 ± 21.41	132.06 ± 22.55	0.569	0.570
DBP (mmHg)	84.48 ± 15.81	81.39 ± 13.01	−1.550	0.123
HR (bpm)	80.27 ± 14.87	78.97 ± 13.81	−0.651	0.516
Smoking (%)	92 (46.9)	88 (39.6)	1.131	0.288
Drinking (%)	38 (19.4)	32 (14.4)	0.923	0.337
Hypertension (%)	96 (49.0)	134 (60.4)	2.724	0.099
Diabetes (%)	38 (19.4)	46 (20.7)	0.058	0.810
Laboratory index				
WBC(×10^9 ^/L)	7.95 (6.40,10.80)	7.10 (5.90,9.60)	2.013	0.044
RBC(×10^12 ^/L)	4.70 (4.40,5.00)	4.40 (4.20,4.70)	4.478	0.000
HGB(g/L)	144.61 ± 13.85	135.68 ± 15.52	−4.363	0.000
PLT(×10^9 ^/L)	259.22 ± 57.28	227.16 ± 53.78	−4.172	0.000
ALT(U/L)	29.55 (18.93,48.24)	23.06 (15.90,41.79)	2.446	0.014
AST(U/L)	33.25 (21.05,145.38)	30.50 (19.80,171.20)	0.755	0.450
Cr(ummol/L)	62.80 (57.30,75.25)	66.20 (55.60,74.60)	−0.612	0.541
BUN(mmol/L)	4.87 (4.24,5.92)	5.22 (4.47,6.17)	−1.673	0.094
Hcy(umol/L)	11.50 (9.18,16.32)	12.40 (9.40,15.70)	−0.422	0.673
UA(umol/L)	311.95 (270.80,382.45)	296.00 (252.20,369.10)	1.549	0.121
Glucose(mmol/L)	6.40 (5.19,8.71)	6.55 (5.12,8.57)	0.213	0.831
TC(mmol/L)	4.99 (4.17,5.87)	4.37 (3.93,4.96)	3.680	0.000
Triglyceride(mmol/L)	1.46 (1.04,2.00)	1.30 (0.99,1.71)	2.081	0.037
HDL-c(mmol/L)	1.09 (0.95,1.26)	1.06 (0.93,1.31)	0.306	0.760
LDL-c(mmol/L)	3.09 (2.51,3.75)	2.66 (2.30,3.06)	3.577	0.000
Lipoprotein a(mg/dl)	18.73 (11.07,36.29)	22.39 (13.26,36.23)	−1.193	0.233
NT-proBNP(pg/ml)	134.50 (46.83,720.00)	322.80 (107.20,1,436.00)	−3.659	0.000
Creatine kinase(U/L)	138.75 (72.70,1,196.30)	138.60 (71.60,818.20)	0.345	0.730
Creatine kinase isoenzyme(ng/ml)	2.94 (1.20,69.39)	2.90 (1.33,48.22)	−0.261	0.794
Troponin I(ng/ml)	0.18 (0.02,22.82)	0.06 (0.03,15.79)	−0.480	0.631
D-dimer(ug/ml)	0.19 (0.12,0.32)	0.24 (0.18,0.39)	−2.671	0.008
Cardiac color ultrasound				
LA(mm)	35.00 (32.00,37.00)	36.00 (33.00,38.00)	−0.884	0.377
LV(mm)	50.00 (47.00,53.00)	49.00 (46.00,52.00)	1.108	0.268
EF(%)	58.00 (46.00,60.00)	58.00 (47.00,60.00)	0.013	0.990
Research index				
RC(mmol/L)	0.69 (0.45,1.01)	0.58 (0.38,0.78)	2.718	0.007
RDW(fL)	39.98 ± 2.43	40.98 ± 2.55	2.889	0.004
BMI(kg/m^2^)	26.76(24.36,29.38)	24.22(22.04,26.89)	4.577	0.000

Note: Abbreviations: SBP, systolic blood pressure; DBP, diastolic blood pressure; HR, heart rate; WBC, white blood cell count; RBC, red blood cell count; HGB, hemoglobin; PLT, platelet count; ALT, alanine aminotransferase; AST, aspartate aminotransferase; Cr, creatinine; BUN, blood urea nitrogen; Hcy, homocysteine; UA, uric acid; TC, total cholesterol; HDL-c, high density lipoprotein cholesterol; LDL-c, low density lipoprotein cholesterol; LA, left atrial size; LV, Left ventricular size; EF, ejection fraction; RC, remnant cholesterol; RDW, red blood cell distribution width; BMI, body mass index.

In summary, early-onset ACS patients differed significantly from late-onset patients in several hematologic and metabolic parameters. These differences may provide insights into distinct pathophysiological mechanisms underlying premature ACS.

### Subgroup analysis of RC, RDW, and BMI in ACS patients

**Figure 3:**
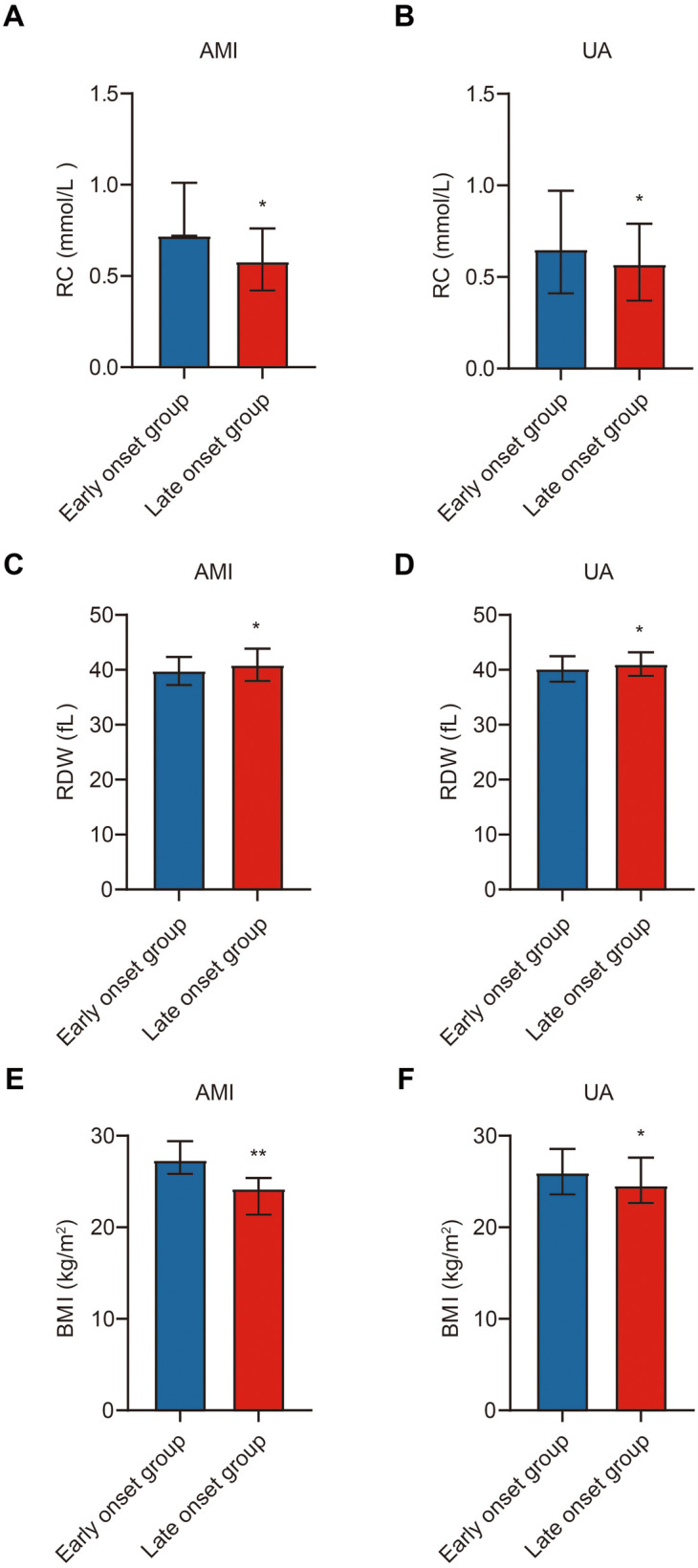
Subgroup comparison of study indicators in ACS patients. Note: **(A)** Comparison of RC between the early-onset and late-onset groups in the AMI subgroup; **(B)** Comparison of RC between the early-onset and late-onset groups in the UA subgroup; **(C)** Comparison of RDW between the early-onset and late-onset groups in the AMI subgroup; **(D)** Comparison of RDW between the early-onset and late-onset groups in the UA subgroup; **(E)** Comparison of BMI between the early-onset and late-onset groups in the AMI subgroup; **(F)** Comparison of BMI between the early-onset and late-onset groups in the UA subgroup. Early-onset group (*n* = 196), late-onset group (*n* = 222). Statistical significance is indicated as *p < 0.05, **p < 0.01.

A detailed subgroup analysis was performed to compare RC, RDW, and BMI between early-onset and late-onset patients within the AMI and UA subgroups. In the AMI subgroup, early-onset patients had significantly higher RC and BMI levels, and lower RDW levels compared to late-onset patients (*p* < 0.05). A similar pattern was observed in the UA subgroup, with early-onset patients again showing elevated RC and BMI and reduced RDW (*p* < 0.05) (Table 2; Figure 3).

**Table 2:** Subgroup comparison.

Variables	Early onset group (AMI)	Late onset group (AMI)	Early onset group (UA)	Late onset group (UA)	*t/Z*	*P* value
	(*n* = 102)	(*n* = 106)	(*n* = 94)	(*n* = 116)		
RC (mmol/L)	0.72 (0.46,1.01)	0.58 (0.42,0.76)			2.415	0.018
			0.65 (0.41,0.97)	0.57 (0.37,0.79)	2.122	0.036
RDW (fL)	39.80 ± 2.56	40.91 ± 2.95			−2.041	0.044
			40.17 ± 2.30	41.04 ± 2.15	−2.001	0.048
BMI (kg/m^2^)	27.34 (25.83,29.41)	24.22 (21.39,25.39)			4.933	0.000
			25.95 (23.59,28.55)	24.56 (22.66,27.61)	1.444	0.049

Note: Abbreviations: RC, remnant cholesterol; RDW, red blood cell distribution width; BMI, body mass index.

These findings further support that RC, BMI, and RDW differ significantly across age-defined ACS groups and may reflect underlying biological distinctions in the pathogenesis of premature vs. late-onset disease.

### Comparison of coronary angiographic features between early-onset and late-onset ACS patients

To further evaluate differences in coronary pathology, angiographic features were compared between early-onset and late-onset ACS patients (Table 3). The late-onset group exhibited a significantly higher incidence of coronary artery calcification (*p* < 0.05) and a greater number of affected vessels (*p* < 0.05). However, no significant difference was observed in Gensini scores between the two groups (*p* > 0.05). Regarding interventional strategies, the early-onset group had a significantly higher intervention rate in the left circumflex artery (LCX) (*p* < 0.05), while no significant differences were found in intervention rates for the left anterior descending artery (LAD) and right coronary artery (RCA) (*p* > 0.05).

**Table 3:** Comparison of coronary angiography.

Variables	Early onset group	Late onset group	*Z/χ*^2^/*t*	*P* value
	(*n* = 196)	(*n* = 222)		
Gensini score	39.50 (23.50,74.75)	42.00 (25.00,70.00)	−0.577	0.564
Coronary calcification (%)	18 (9.2)	46 (20.7)	5.343	0.021
Lesion count	1.40 ± 0.77	1.61 ± 0.73	2.070	0.040
Intervention vessels				
LAD (%)	114 (58.2)	110 (49.5)	1.553	0.213
LCX (%)	46 (23.5)	18 (8.1)	9.472	0.002
RCA (%)	82 (41.8)	80 (36.0)	0.738	0.390

Note: Abbreviations: LAD, left anterior descending; LCX, left circumflex; RCA, right coronary artery.

In summary, late-onset patients exhibited more extensive coronary calcification and multivessel disease, while early-onset patients were more likely to undergo LCX intervention. These findings suggest that age of onset may influence coronary lesion characteristics and treatment patterns, with potential implications for individualized therapy.

### Evaluation and clinical value of combined prediction of premature ACS using RC, RDW, and BMI

To assess the predictive value of RC, RDW, and BMI for premature ACS, correlation analysis was first conducted. RC (*r* = 0.527, *p* < 0.0001) and BMI (*r* = 0.574, *p* < 0.0001) showed significant positive correlations, while RDW exhibited a strong negative correlation with premature ACS (*r* = −0.589, *p* < 0.0001) (Table 4). Subsequently, multivariate logistic regression identified all three variables—RC (*p* = 0.0013), RDW (*p* < 0.0001), and BMI (*p* < 0.0001)—as independent risk factors for premature ACS (Table 5).

**Table 4:** Relevance analysis.

Indicators	*r*	*P* value
RC	0.527	<0.0001
RDW	−0.589	<0.0001
BMI	0.574	<0.0001

Note: Abbreviations: RC, remnant cholesterol; RDW, red blood cell distribution width; BMI, body mass index.

**Table 5:** Logistic regression analysis.

Variables	Parameter	SE	95%CI	Z	*P*
RC	4.992	1.557	2.039–8.193	3.206	0.0013
RDW	−1.247	0.2382	−1.758−0.8172	5.235	<0.0001
BMI	0.8157	0.1530	0.5359–1.141	5.332	<0.0001

Note: Abbreviations: RC, remnant cholesterol; RDW, red blood cell distribution width; BMI, body mass index.

**Figure 4:**
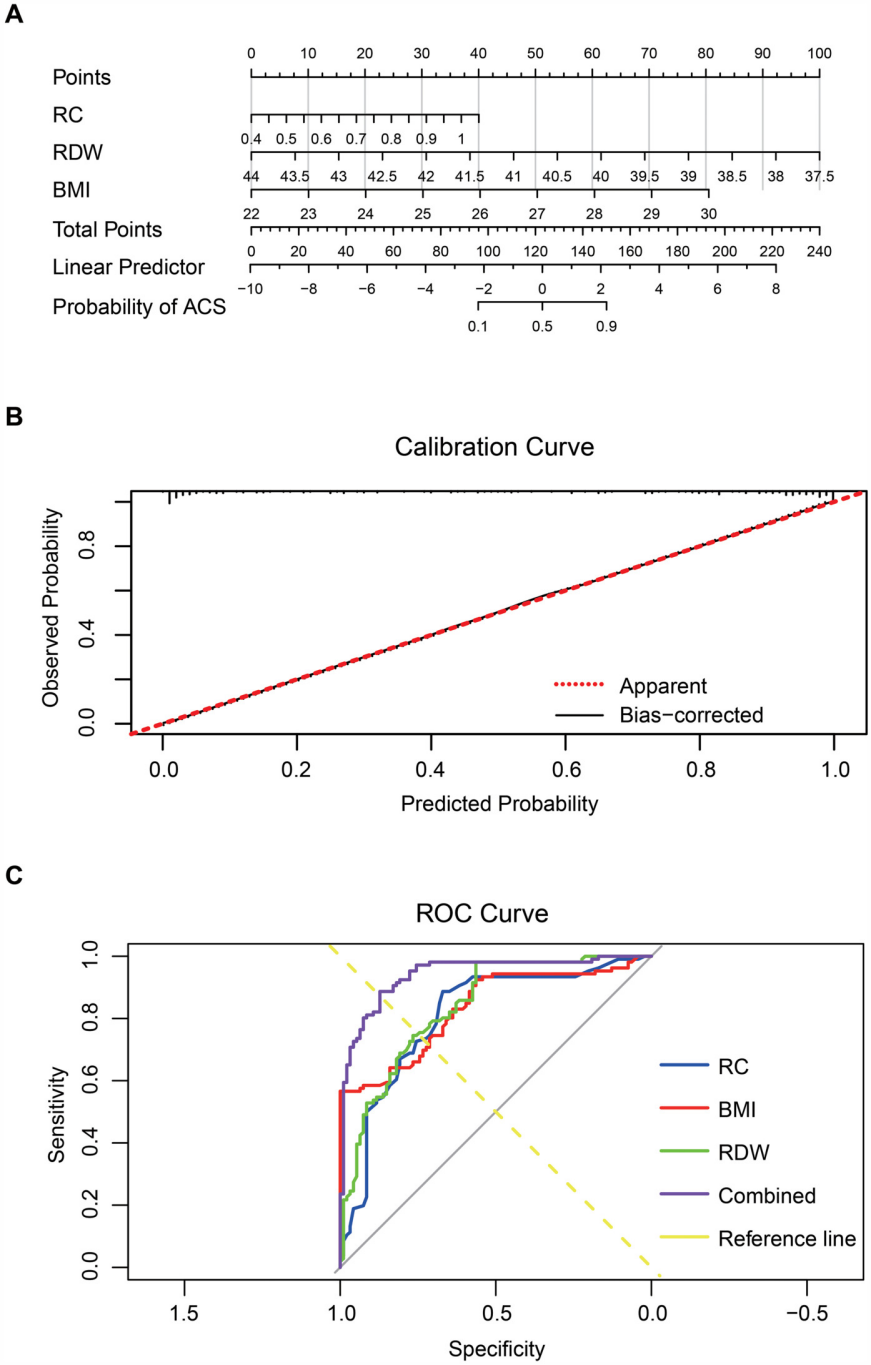
RC, RDW, and BMI combined to predict premature ACS. Note: **(A)** Nomogram constructed using RC, RDW, and BMI; **(B)** Calibration curve assessing the nomogram model's predictive accuracy; **(C)** ROC curves of RC, RDW, BMI, and the combined multivariable model.

Based on these results, a nomogram model was constructed to provide quantitative risk estimation. Among the predictors, RC contributed the widest point range, with higher values associated with greater risk. RDW and BMI also significantly impacted risk: lower RDW and higher BMI corresponded to increased probability of premature ACS. The total nomogram score ranged from 0 to 240, with higher scores indicating greater risk (Figure 4A). The model demonstrated excellent discriminative ability, achieving a C-index of 0.941. Calibration analysis based on 1,000 bootstrap resamples showed strong agreement between predicted and observed outcomes (Figure 4B). ROC analysis revealed that RC, RDW, and BMI individually achieved AUCs of 0.807, 0.837, and 0.833, respectively. The combined model produced an AUC of 0.941, significantly outperforming individual markers. The ROC curve of the multivariate model closely approached the upper-left corner, reflecting superior sensitivity, specificity, and overall diagnostic accuracy (Figure 4C).

Collectively, these results indicate that integrating RC, RDW, and BMI enhances the early identification of individuals at high risk for premature ACS. This multifactorial approach offers important clinical value for precision diagnosis and targeted prevention.

## Discussion

ACS remains one of the leading causes of cardiovascular morbidity and mortality worldwide, posing a persistent threat to global public health (3). The underlying pathology involves atherosclerotic plaque rupture, erosion, or thrombosis, leading to acute myocardial ischemia (1). Despite improvements in diagnostic and interventional strategies, early identification of high-risk individuals—particularly those with premature ACS—remains a clinical challenge. Many young patients lack typical symptoms or present with normal LDL-C levels, making timely diagnosis difficult (4). The main findings of this study are as follows. First, RC and BMI levels were significantly higher in patients with premature ACS compared to those with late-onset ACS, while RDW was significantly lower. RC and BMI showed positive correlations with premature ACS, whereas RDW was negatively correlated. Second, all three markers—RC, RDW, and BMI—were identified as independent risk factors for premature ACS. Third, the combination of these markers significantly improved diagnostic performance, supporting the development of a novel multifactorial early diagnostic model. Clinically, these routinely available and cost-effective biomarkers may assist in identifying high-risk individuals earlier, facilitating timely intervention and improved patient outcomes.

These results are largely consistent with previous research, while also revealing some differences. Prior studies have established a strong link between RC and atherosclerosis or cardiovascular events (10, 20). Our findings further demonstrate that RC levels are elevated in premature ACS and confirm RC as an independent risk factor, aligning with results reported by Cordero et al. (9). Mechanistically, RC may promote the progression of ACS through inflammatory pathways and lipid deposition, independent of traditional markers such as LDL-C. The relationship between RDW and ACS has also been well-documented, with elevated RDW generally associated with worse cardiovascular outcomes (21). In contrast, our study observed a negative correlation between RDW and premature ACS. This discrepancy may be attributed to variations in inflammatory status, oxidative stress, or erythropoietic activity unique to younger ACS patients. Additionally, the role of BMI in cardiovascular risk is well-supported (22), and our findings further highlight its contribution to premature ACS, underscoring the growing importance of obesity as a modifiable risk factor.

Recent studies have highlighted the significance of integrating metabolic and inflammatory markers in assessing the risk and severity of ACS. Hori et al. demonstrated that triglyceride levels and BMI are independent determinants of sdLDL-C in ACS patients, with those exhibiting both high triglyceride and high BMI levels showing approximately threefold higher sdLDL-C levels compared to those with lower values (14). Similarly, Yuan et al. found that combining the NLR with the LDL-C/HDL-C ratio provided superior predictive value for the severity of coronary lesions in ACS patients. Their study also identified significant associations between BMI, RDW, and coronary lesion severity, underscoring the potential of integrated biomarker models in ACS risk stratification (15). These findings align with our results, suggesting that the combined assessment of RC, RDW, and BMI could enhance early detection and risk stratification for premature ACS.

The negative correlation between RDW and premature ACS observed in this study was unexpected. Previous studies have generally reported that elevated RDW is associated with increased severity and poor prognosis in ACS patients. In contrast, our findings show significantly lower RDW levels in patients with premature ACS. Several possible explanations may account for this discrepancy. First, our study population consisted exclusively of ACS patients, and RDW baseline levels may differ across age groups and pathological stages. Second, RDW may be influenced by diverse factors such as chronic inflammation, oxidative stress, and bone marrow activity, which could vary in premature ACS. For example, changes in RDW may reflect acute-phase red blood cell deformability, endothelial damage, or hemodynamic alterations. These differences highlight the complexity of RDW behavior in specific clinical contexts and indicate that further research is needed to elucidate its role in premature ACS.

This study offers new insights into the early diagnosis and risk stratification of premature ACS. RC, RDW, and BMI are simple, low-cost biomarkers that can be readily obtained through routine clinical assessments. As an independent risk factor, RC provides additional diagnostic information, especially in patients with well-controlled LDL-C levels. RDW and BMI also reflect systemic inflammation and metabolic status, contributing to a more comprehensive risk evaluation. Their clinical utility lies in their accessibility and ability to identify high-risk individuals before symptom onset. We recommend incorporating these markers into standard cardiovascular assessments to improve early detection and intervention.

From a real-world clinical perspective, the combined use of RC, RDW, and BMI offers a cost-effective and accessible tool for early risk assessment of premature ACS. These biomarkers are routinely collected during standard blood tests or physical examinations, without the need for specialized equipment or additional cost. In particular, residual cholesterol can be calculated indirectly from existing lipid panels (TC—HDL-C—LDL-C), making it feasible to implement even in primary care settings. This is especially relevant in younger patients who may present atypically or lack traditional risk factors, where early warning signs are often missed. Our findings support the idea that integrating these markers into routine cardiovascular screening protocols could enhance the early identification of high-risk individuals and facilitate timely preventive strategies, potentially reducing the burden of ACS in younger populations. Moreover, as electronic health records and AI-driven risk tools evolve, these simple parameters could be easily incorporated into predictive algorithms for wider clinical use.

The limitations of this study are as follows: (1) although the total sample size of 418 patients is relatively adequate compared to similar biomarker studies, it remains modest given the high prevalence and clinical heterogeneity of ACS. A larger cohort would strengthen the statistical power and generalizability of the findings, particularly for subgroup analyses. (2) the study was conducted in a single tertiary center, which may introduce selection bias and limit external validity. (3) important covariates such as physical activity, dietary habits, and family history were not available, which could confound the observed associations. (4) This study did not further analyze the relationships between these biomarkers and specific ACS subtypes, such as the differences between STEMI and NSTEMI patients. Future studies should be based on larger sample sizes and incorporate more clinical information and cardiac imaging data to further validate the roles of these biomarkers in different ACS subtypes.

Future research should explore the biological mechanisms underlying the associations between RC, RDW, BMI, and premature ACS. In particular, the inverse relationship between RDW and early-onset ACS warrants further investigation through experimental and longitudinal studies. Advances in genomics, proteomics, and high-throughput screening may also help refine predictive models and enable more personalized approaches to cardiovascular risk assessment. Ultimately, integrating diverse biomarker domains—lipid metabolism, inflammation, and anthropometry—may enhance our ability to identify and manage individuals at risk of premature ACS.

## Conclusion

**Figure 5:**
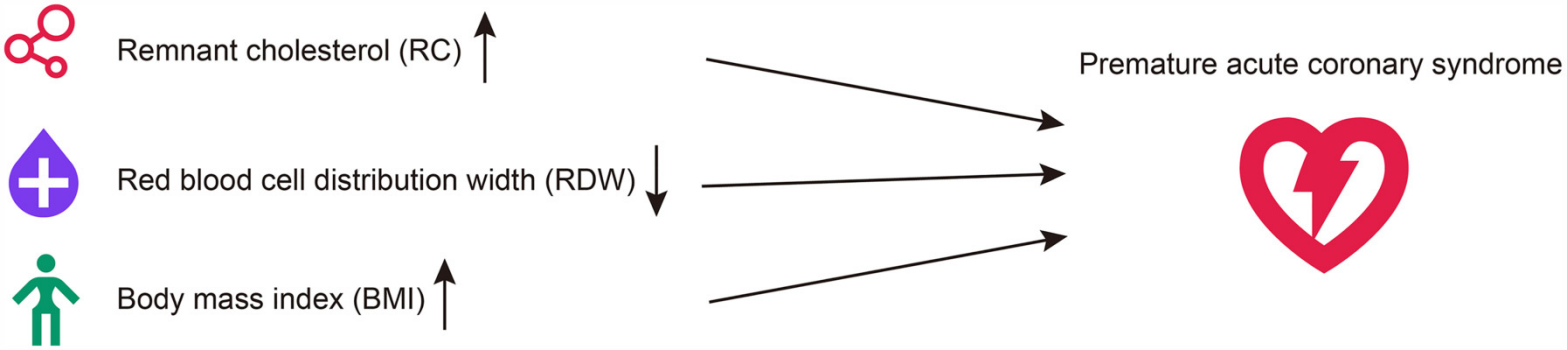
Mechanistic diagram illustrating how RC, RDW, and BMI contribute to the prediction of premature ACS.

This study investigates the predictive value of RC, RDW, and BMI in patients with premature ACS. The findings demonstrate that RC and BMI are positively associated with premature ACS, whereas RDW shows a negative correlation (Figure 5). All three factors were identified as independent risk factors. Furthermore, logistic regression and ROC curve analyses further confirmed that the combined use of RC, RDW, and BMI significantly improved diagnostic performance, enhancing both diagnostic sensitivity and specificity for the early detection of premature ACS.

## Data Availability

The raw data supporting the conclusions of this article will be made available by the authors, without undue reservation.
